# The use of intravenous immunoglobulin as a rescue therapy for refractory parainfectious leprosy-related neuritis: a case series^☆^

**DOI:** 10.1016/j.abd.2023.09.007

**Published:** 2024-06-03

**Authors:** João Guilherme Pessôa Léo, Camila Bocchi Siqueira, Jorgeth de Oliveira Carneiro da Motta, Ingrid Faber de Vasconcellos, Yuna Ribeiro de Araujo, Felipe Von Glehn, Patrícia Shu Kurizky, Ciro Martins Gomes, Cláudia Porto, Maria Stella Cochrane Feitosa

**Affiliations:** a Department of Dermatology, Hospital Universitário de Brasília, Brasília, DF, Brazil; b Department of Neurology, Hospital Universitário de Brasília, Brasília, DF, Brazil; c Department of Dermatology, Hospital Regional da Asa Norte, Brasília, DF, Brazil

Dear Editor,

Leprosy-related parainfectious immune-mediated damage to peripheral nerves can lead to permanent physical disabilities.^1^ Leprosy Neuritis (LN) is an acute inflammation of the peripheral nerves that occurs in some cases of leprosy. The current treatment of choice is the use of immunosuppressive doses of corticosteroids. There is growing evidence that corticosteroids may not be sufficient for a considerable number of leprosy patients experiencing refractory LN.^2^

We report here a case series of patients diagnosed with LN refractory to systemic corticosteroids who were treated with Intravenous Immunoglobulin (IVIg) as a rescue therapy for recovering the function of peripheral nerves.

Patients were evaluated at the Dermatology Outpatient Clinic University Hospital of Brasília, Brazil from 2016 to 2022. All patients received IVIg for the treatment of refractory leprosy reactions. The study was approved by the Research Ethics Committee of the Faculty of Medicine of the University of Brasília (CEP-FM/UnB) (72312117.4.0000.5558).

All patients had a diagnosis of leprosy confirmed by 2 board-certified dermatologists following the World Health Organization (WHO) criteria supported by polymerase chain reaction as described elsewhere.^3^ Prior to the prescription of IVIg, all patients were evaluated by a board-certified neurologist. Detailed clinical and laboratory evaluations were performed, including searches for tuberculosis, sexually transmitted infections and American trypanosomiasis. Those conditions represented exclusion criteria for the use of IVIg. We used the Universal Pain Assessment Tool (UPAT) for pain evaluation, which has a scale from 1 to 10, and the WHO Disability Grading System, which has a scale from 0 to 2. Sensitivity tests were performed using the Semmes-Weinstein monofilaments according to the recommendations of the Brazil Ministry of Health. Electroneuromyography was performed 1-week before and 1-month after the 5^th^ infusion cycle of IVIg.

Six patients were enrolled (Table 1). In only two cases, slit skin smears and PCR results were both negative. Three patients were classified as having leprosy relapse (Table 1). All patients had a diagnosis of leprosy-related neuritis with acute nerve function deterioration diagnosed by serial simplified neurological evaluation and by specialized neurological evaluation.

**Table 1:** Demographic and clinical characteristics of the 6 leprosy patients experiencing refractory leprosy-related neuritis who received intravenous immunoglobulin.

N	Age	Sex	Leprosy Classification	Electroneuromyography pattern before IVIg	Slit Skin Smear	Polymerase Chain Reaction	Relapse	Current Leprosy Treatment	Leprosy reactions^a^
1	70	M	Pure Neural Leprosy	Sensitive > Motor demyelinating pattern	Negative	Negative	No	WHO-MDT for 12-months	Classic type 1 reaction
2	34	F	BB	Sensitive > Motor demyelinating pattern	Positive	Positive	Yes	Alternative MDT Rifampicin, Minociclin and Clofazimine for 12-months	Classic type 1 reaction
3	48	F	BB	Sensitive > Motor axonal pattern	Negative	Positive	Yes	Alternative MDT with Rifampicin, Moxifloxacin and Clofazimine for 24-months	Type 1 and 2 reactions (concomitant use of thalidomide)
4	58	M	Pure Neural Leprosy	Sensitive > motor demyelinating pattern with axonal components	Negative	Negative	No	WHO-MDT for 12-months	Neuritis without other symphtoms
5	43	F	BB	Sensitive > motor demyelinating pattern.	Negative	Positive	Yes	Alternative MDT Rifampicin, Minociclin and Ofloxacin for 12-months	Classic Type 1 reaction
6	24	F	BB	Sensitive demyelinating pattern	Negative	Positive	No	WHO-MDT for 12-months	Classic Type 1 reaction

N, Patient number; IVIg, Intravenous Immunoglobulin; M, Male; F, Female; BB, Borderline-Borderline leprosy form; WHO, World Health Organization; MDT, Multidrug Therapy.

a Patients who also presented cutaneous reactions were classified as having a Type 1 reaction while patients who presented only with neuritis, considered by some authors a spectrum of a Type 1 reaction, were clearly labeled as having neuritis without other symptoms.

All patients had received more than 3 cycles of prednisone for at least 6 months at a minimal starting dose of 1 mg/kg/day (Table 2). One patient also received high doses of IV methylprednisolone (N4). Four patients underwent surgical decompression of the affected peripheral nerves (neurolysis), and 3 also underwent transposition of the peripheral nerves. All surgical procedures occurred at least 2 months prior to the 1^st^ IVIg infusion. They received adjuvant treatment with either pregabalin, gabapentin, duloxetine or opioids (codeine or methadone).

**Table 2:** Therapeutics and clinical response of the 6 leprosy cases experiencing refractory leprosy-related neuritis that received intravenous immunoglobulin.

N	Period from diagnosis to IVIg (months)	Other medication for neuropathic pain	Number of IVIg cycles	Neurolysis/Transposition of peripheral nerves	Degree of physical disability before/after IVIg	UPAT before/after IVIg	Comments
1	6	Duloxetine and Pregabaline	5	Yes/No	2/2	7/1	Pain improvement, improvement of palmar and plantar sensitivity and maintenance of mobile left ulnar claw
2	12	Pregabaline	5	Yes/Yes	2/2	8/2	Pain improvement
3	60	Duloxetine, Gabapentine, Codeine and Metadone	5	Yes/Yes	2/2	10/4	Pain improvement for only 20 days after IVIg infusion, manutention of palmar and plantar hypoesthesia, maintenance of mobile left ulnar claw
4	12	Pregabaline	8	No/No	2/1	7/1	Pain improvement, improvement of palmar and plantar sensitivity and almost complete recovery of foot dorsiflexion
5	12	Gabapentine, Duloxetine, Oxcarbazepine	5	Yes/Yes	1/1	5/5	Maintenance of bilateral paresia of superior limbs
6	6	No	5	No/No	1/1	7/1	Pain improvement and improvement of palmar sensitivity

N, Patient number; UPAT, Universal Pain Assessment Tool; IVIg, Intravenous Immunoglobulin.

Despite therapy, the disease in all patients evolved with worsening pain complaints and sensitivity, and motor function impairment. All patients already had adverse events related to the use of corticosteroids, including Cushingoid facies and high blood pressure. Patient 3 experienced a rapid evolution of cataracts. One patient also presented with opioid use disorder.

The induction dose of IVIg (Gamunex, Grifols Therapeutics LLC, Los Angeles, USA) was 2 g/kg divided across five days for all infusion cycles. Infusions were repeated for 5 cycles at monthly intervals. Additional doses were prescribed depending on the clinical response. All patients started immunoglobulin infusions with adjuvant 1 mg/kg/day prednisone or equivalent doses, and dosages were tapered weekly according to the response.

The median time from diagnosis to the first IVIg infusion was 12 months (range = 6–60). All patients (including relapse cases) received the first multidrug therapy dose on the same day of leprosy diagnosis. All patients were followed up for at least 12 months after the first IVIg cycle. In most cases, a dramatic reduction in pain and an evident improvement in sensitivity and motor functions were achieved (Table 2). Patients reported a median UPAT of 7 (range = 5–10) before and a median value of 1.5 (range = 1–5) after the 5^th^ IVIg infusion. A sustained response after 5 doses of IVIg was presented in 4 patients (N1, 2, 5 and 6), even 6 months after the last infusion. One patient needed a total of 8 infusions to achieve remission (N4). Patient N3, the only one who presented a concomitant type 2 reaction and was using thalidomide, presented pain improvement for only 20 days with apparently no improvement in sensitivity and motor functions. This patient started IVIg therapy considerably later than the others, and the disease in both patients 3 and 4 evolved with considerably greater axonal neuropathy patterns demonstrated by electroneuromyography than that in other patients (Table 1).

According to the UPAT scale, 4 of the 6 patients experienced significant and long-lasting pain relief, while 1 remained stable (N5). Except for patient N3, all patients presented variable improvements in conduction velocity and/or amplitude of potentials in the electroneuromyography exam performed 1-month after the 5^th^ IVIg infusion.

IVIg is a well-established treatment for demyelinating neuropathies. IVIg has a strong anti-inflammatory effect. Previous studies revealed that the levels of IL-1β, TNF, IL-6 TGF-β, and IL-17 are elevated in the sera and blood of leprosy patients experiencing LN.^4^ The significant effect of IVIg on pain relief could be linked to a reduction in inflammation and damage to myelin and nerves.

In two patients, slit skin smears and PCR did not reveal *M.* leprae. A systematic exclusion of other causes of peripheral neuropathy is mandatory. An important limitation related to the treatment with IVIg is that there are different methods used to produce different IVIg brands. Previous studies have shown that different brands can have different clinical effects.^5^

IVIg also acts by reducing the levels of proinflammatory subsets of peripheral blood monocytes (CD14+CD16++).^6^ Additionally, in a mouse model of multiple sclerosis, IVIg abrogated disease progression by inhibiting the proinflammatory Th17 pathway.^7^ Apparently, IL17 has an important role in LN.^2^

We can conclude that IVIg is an interesting option for the treatment of refractory LN. It can reduce the deleterious effects of high doses of corticosteroids. There are currently no alternative approaches for the treatment of LN that does not respond to corticosteroids. The long-term results of the medication and the safety of IVIg for LN must be urgently assessed by clinical trials.

## Financial support

FUNADERM ‒ Fundo de Apoio à Dermatologia.

## Authors’ contributions

João Guilherme Pessôa Léo: Drafting and editing of the manuscript or critical review of important intellectual content.

Camila Bocchi Siqueira: Design and planning of the study.

Jorgeth de Oliveira Carneiro da Motta: Collection, analysis and interpretation of data.

Ingrid Faber de Vasconcellos: Drafting and editing of the manuscript or critical review of important intellectual content.

Yuna Ribeiro de Araujo: Intellectual participation in the propaedeutic and/or therapeutic conduct of the studied cases.

Felipe Von Glehn: Drafting and editing of the manuscript or critical review of important intellectual content.

Patrícia Shu Kurizky: Collection, analysis and interpretation of data and critical review of the literature.

Ciro Martins Gomes: Statistical analysis; drafting and editing of the manuscript or critical review of important intellectual content; critical review of the literature; approval of the final version of the manuscript.

Cláudia Porto: Drafting and editing of the manuscript or critical review of important intellectual content.

Maria Stella Cochrane Feitosa: Design and planning of the study; data collection, or analysis and interpretation of data; collection, analysis and interpretation of data; effective participation in research orientation; approval of the final version of the manuscript.

## Conflicts of interest

None declared.
